# Identification of New Splice Sites Used for Generation of *rev* Transcripts in Human Immunodeficiency Virus Type 1 Subtype C Primary Isolates

**DOI:** 10.1371/journal.pone.0030574

**Published:** 2012-02-17

**Authors:** Elena Delgado, Cristina Carrera, Paloma Nebreda, Aurora Fernández-García, Milagros Pinilla, Valentina García, Lucía Pérez-Álvarez, Michael M. Thomson

**Affiliations:** Centro Nacional de Microbiología, Instituto de Salud Carlos III, Majadahonda, Madrid, Spain; Institut National de la Santé et de la Recherche Médicale, France

## Abstract

The HIV-1 primary transcript undergoes a complex splicing process by which more than 40 different spliced RNAs are generated. One of the factors contributing to HIV-1 splicing complexity is the multiplicity of 3′ splice sites (3'ss) used for generation of *rev* RNAs, with two 3'ss, A4a and A4b, being most commonly used, a third site, A4c, used less frequently, and two additional sites, A4d and A4e, reported in only two and one isolates, respectively. HIV-1 splicing has been analyzed mostly in subtype B isolates, and data on other group M clades are lacking. Here we examine splice site usage in three primary isolates of subtype C, the most prevalent clade in the HIV-1 pandemic, by using an *in vitro* infection assay of peripheral blood mononuclear cells. Viral spliced RNAs were identified by RT-PCR amplification using a fluorescently-labeled primer and software analyses and by cloning and sequencing the amplified products. The results revealed that splice site usage for generation of *rev* transcripts in subtype C differs from that reported for subtype B, with most *rev* RNAs using two previously unreported 3'ss, one located 7 nucleotides upstream of 3'ss A4a, designated A4f, preferentially used by two isolates, and another located 14 nucleotides upstream of 3'ss A4c, designated A4g, preferentially used by the third isolate. A new 5′ splice site, designated D2a, was also identified in one virus. Usage of the newly identified splice sites is consistent with sequence features commonly found in subtype C viruses. These results show that splice site usage may differ between HIV-1 subtypes.

## Introduction

**Figure 1:**
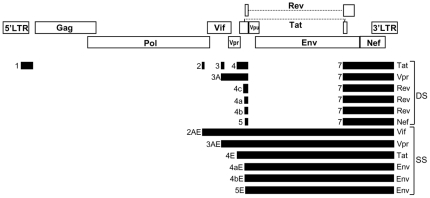
Schematic representation of HIV-1 splicing. Open reading frames are shown as open boxes and exons as black bars. Exons are named as previously [1], [2]. All spliced transcripts incorporate exon 1. Optionally, noncoding exons 2 or 3 or both can be incorporated into *tat*, *rev*, *nef*, or *env* transcripts, and exon 2 into *vpr* transcripts. Proteins encoded in spliced RNAs are indicated on the right of the 3′ exon.

All HIV-1 RNAs are transcribed from a single promoter at the 5′ long terminal repeat, and their relative expression is regulated through alternative splicing. According to the splicing events used for their generation, HIV-1 RNAs can be assigned to three categories: 1) unspliced RNA, coding for Gag and Pol; 2) singly spliced (SS) transcripts, which code for Env, Vpu, Vif, Vpr, and a truncated form of Tat; and 3) doubly spliced (DS) transcripts, which code for Tat, Rev, Nef, and Vpr. Four 5′ splice sites (5'ss) and nine 3′ splice sites (3'ss) (including three 3'ss used by *rev* RNAs, A4a, A4b, and A4c) are commonly used by HIV-1, generating more than 40 different transcripts [1], [2] (Fig. 1). Additionally, multiple other splice sites are used infrequently [1], [3]–[10]. Most HIV-1 splice sites exhibit suboptimal efficiencies [11]–[15], which allow for regulation of their relative usage by the action of cellular splice regulatory factors binding to splice enhancer and suppressor elements in the HIV-1 genome [16].

Previous studies on HIV-1 splicing have been done almost exclusively using subtype B viruses, usually T-cell line-adapted isolates. To our knowledge, non-subtype B viruses reported to be analyzed for splicing patterns are limited to two group O viruses [8], [17]. Here we analyze splice site usage by primary isolates of subtype C, the most prevalent clade in the HIV-1 pandemic [18], using an *in vitro* infection assay of peripheral blood mononuclear cells (PBMCs).

## Materials and Methods

Three subtype C primary isolates, X1702-3, X1936, and X2363-2 [19], [20], were used for infection of PBMCs, obtained from healthy donors, who gave their written informed consent. For each isolate, infection assays were done in triplicate using PBMCs from three different donors. The subtype B isolate NL4-3 was used as control in one of the assays. PBMCs were prestimulated with phytohemagglutinin and interleukin-2 for three days and exposed to virus at a multiplicity of infection of 0.1 50% tissue culture infectious dose (TCID_50_) per cell for 2 h, followed by two washes with phosphate-buffered saline. Cells were collected on days 1, 2, 3, 4, and 7 postinfection and total RNA was extracted. HIV-1 splicing patterns were analyzed through RT-PCR followed by nested PCR, using primers recognizing sequences in the outermost exons common to either all DS or SS HIV-1 RNAs, yielding amplified products of different sizes according to the splice sites used for generation of the transcripts. Reagents and PCR conditions were similar to those described previously [10], except that in the nested PCR 15 cycles were used, the sense primer was US22 [CTCGACGCAGGACTCGGCTTGC, HXB2 nucleotides (nt) 685–706], and for DS RNAs the antisense primer was TRN-AS (CGGTGGTAGCTGAARAGGCACAG, HXB2 nt 8511–8533). US22 was 5′-labeled with VIC fluorophore, which allowed for analysis of the amplified products electrophoresed in an automated sequencer by using GeneMapper software program (Applied Biosystems, Carlsbad, CA), which can accurately determine sizes of PCR products by running a size standard labeled with a different fluorophore in the same capillary and quantify them by measuring peak areas. Identification of PCR products with sizes different from those expected by the use of known splice sites was done through TA cloning and sequencing of the amplified products.

## Results

**Figure 2:**
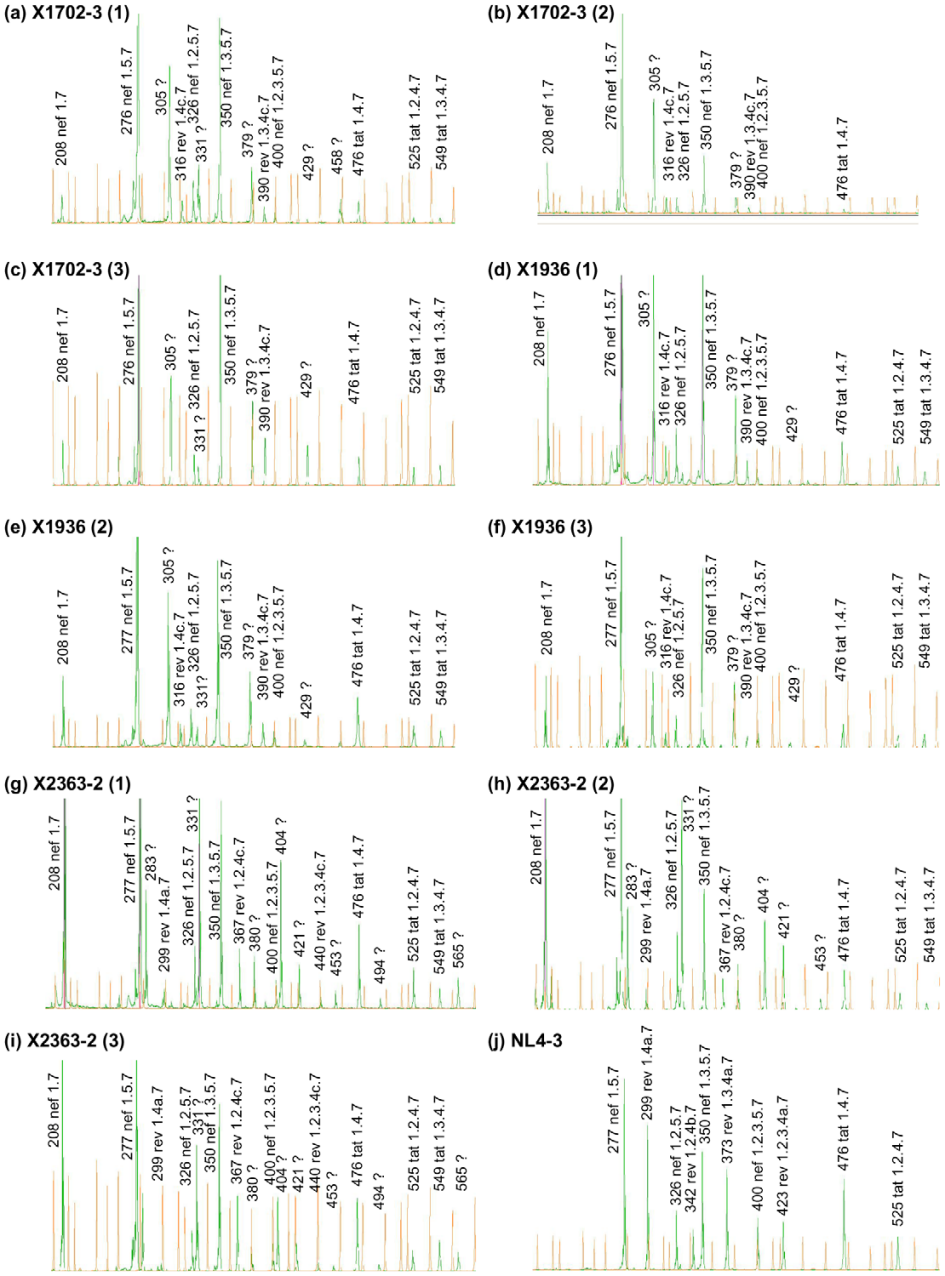
GeneMapper analyses of DS RNAs expressed by three HIV-1 subtype C primary isolates in PBMCs. Green peaks represent PCR products and orange peaks represent size standards. Size of PCR product, encoded gene, and exon composition (named as in previous studies [1], [2]) predicted according to the size of the PCR product are shown on top or on the side of each peak. Peaks whose sizes do not match HIV-1 transcripts using previously reported splice sites are marked with interrogation signs. For each subtype C virus, three GeneMapper analyses are shown, corresponding to infections using PBMCs from three different donors.

GeneMapper analyses revealed that most peaks derived from spliced transcripts in the three subtype C isolates corresponded to sizes expected for HIV-1 transcripts using previously reported splice sites. However in all three viruses, peaks with unexpected sizes were detected among DS transcripts (Fig. 2). In X1702-3 and X1936, three peaks were 7 nt longer than predicted for *rev* transcripts using A4a (1.4a.7, 1.3.4a.7, and 1.2.3.4a.7). Interestingly, in both viruses, transcripts using A4a and A4b, the most common 3'ss used for *rev* RNA generation in subtype B isolates, were not detected. In X2363-2, peaks with sizes 14 nt longer than those corresponding to *rev* transcripts using A4c (1.4c.7, 1.2.4c.7, and 1.3.4c.7) were detected. In NL4-3, all peaks corresponded to sizes expected from the usage of known splice sites (Fig. 2j).

**Figure 3:**
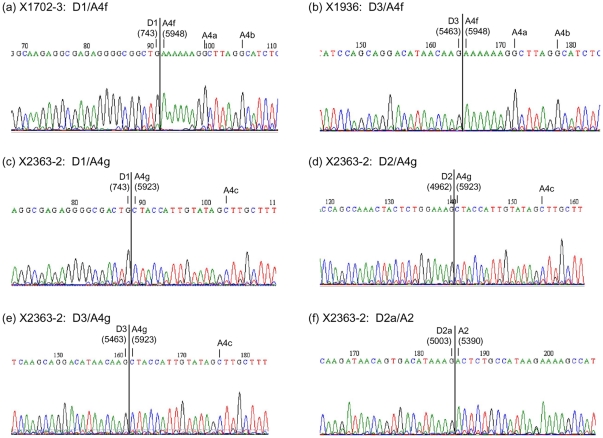
Sequence electropherograms of splice junctions newly identified in subtype C isolates. Splice junctions are shown as vertical lines. 5′ and 3′ splice sites involved in splicing, named as in previous studies [1], [2] and in this study (see main text), are signaled, with nucleotide positions in the HXB2 genome in parentheses. Nearby splice sites are also indicated.

Since most peaks with unexpected sizes were close to those predicted for known *rev* transcripts, and those corresponding to RNAs using 3'ss A4a and A4b were either undetected or relatively weak, we suspected that the unidentified peaks corresponded to *rev* transcripts using previously unreported splice sites. To examine this possibility, nested PCRs using the antisense primer TatRev-AS (GCTTCTTCCTGCCATAGGAGATGC, HXB2 nt 5961–5984) recognizing a sequence downstream of A4b and upstream of A5, able to amplify all known *rev* transcripts, in addition to *tat* and *vpr* (but not *nef*) RNAs, were done using RT-PCR products derived from DS transcripts from PBMCs collected on day 2 postinfection. In all three subtype C viruses, the analyses of sequences of the cloned products revealed the preferential usage of previously unreported 3'ss for generation of *rev* RNAs located at positions in the HIV-1 genome consistent with peaks detected with GeneMapper (Fig. 3, Table 1). In X1702-3 and X1936, *rev* RNAs preferentially used a 3'ss at HXB2 position 5948, 7 nt upstream of A4a, which was designated A4f (named consecutively after A4d, identified in one isolate of subtype B and one of group O, and A4e, identified in a group O virus [8]). A4f was used in 20 (90.9%) of 22 *rev* clones in X1702-3 and in 18 (94.7%) of 19 *rev* clones in X1936, with the remaining *rev* transcripts using A4c. In X2363-2, all 12 analyzed *rev* clones used a 3'ss at HXB2 position 5923, 14 nt upstream of A4c, which was designated A4g (splicing at this site does not create a new open reading frame, since there is no AUG between it and the Rev initiation codon). One clone of X2363-2 contained three noncoding exons upstream of A4g, corresponding to exon 1, a second exon 91 nt long using 3'ss A1 and a newly identified 5'ss at HXB2 position 5003, 41 nt downstream of 5'ss D2 (which was designated D2a), and exon 3. The proportion of *rev* transcripts using A4f in X1702-3 and X1936 and A4g in X2363, as determined by clone sequencing, was generally consistent with quantification of peak areas in GeneMapper analyses (Table 2). Sequencing of clones of PCR products derived from SS RNAs also revealed the usage of A4f in X1702-3 and X1936 and of A4g in X2363 (results not shown).

**Table 1:** Exon composition of clones derived from DS *rev* and *tat* RNAs expressed by three subtype C isolates*.

	*rev*								*tat*	
Isolate	1.4f	1.3.4f	1.2.3.4f	1.4c	1.4g	1.2.4g	1.3.4g	1.2a.3.4g	1.4	Total
X1702-3	15	5		2					1	23
X1936	11	5	2	1					2	21
X2363-2					9	1	1	1		12

Exons 1, 2, 3, 4c, and 4 are named as in previous studies [1], [2]. Exons 4f and 4g designate those using newly identified 3'ss A4f and A4g, respectively, and exon 2a designates that using 3'ss A1 and newly identified 5'ss D2a (see main text). All transcripts are assumed to include exon 7 through splicing from 5'ss D4 to 3'ss A7. However this splice junction was not sequenced, since the antisense PCR primer anneals upstream of D4. *nef* RNAs were not amplified because the antisense PCR primer recognizes a sequence upstream of 3'ss A5.

**Table 2:** Relative expression of *rev* RNAs in subtype C viruses according to peak areas in GeneMapper analyses.

Isolate	PCR product size	Exon composition	% total *rev* RNA peak area	% *rev* clones
X1702-3^1^	305	1.4f.7	70.3	68.2
	316	1.4c.7	7.1	9.1
	379^2^	1.3.4f.7	18.0	22.7
	390	1.2.4c.7	3.9	-
	428	1.2.3.4f.7	0.8	-
X1936^1^	305	1.4f.7	52.2	57.9
	316	1.4c.7	8.6	5.3
	379^2^	1.3.4f.7	27.4	26.3
	390	1.2.4c.7	9.0	-
	428	1.2.3.4f.7	2.7	10.5
X2363-2	299	1.4a.7	2.6	-
	305	1.4f.7	0.8	-
	331	1.4g.7	50.1	75.0
	367^3^	1.2.4c.7	9.1	-
	380^2^	1.2.4g.7	6.7	8.3
	390	1.3.4c.7	0.7	-
	404	1.3.4g.7	19.5	8.3
	421	1.2a.4g.7	5.8	-
	440	1.2.3.4c.7	1.6	-
	454	1.2.3.4g.7	2.0	-
	494	1.2a.3.4g.7	1.1	8.3

Results correspond to peaks shown in Fig. 2, and are shown as % of individual peak areas relative to the sum of peak areas of all *rev* RNA-derived products. Percentages at the column on the right correspond to the cloned and sequenced *rev* RNA-derived amplicons (Table 1).

1 A small 331 nt peak, coincident with that of 1.4g.7 *rev* RNA, was seen in X1702-3 and X1936 (Fig. 2). However, nested PCR using an antisense primer specific for *rev*, *tat* and *vpr* RNAs failed to detect 1.4g.7 *rev* RNA in these isolates.

2 Nested PCR with primers recognizing exons 2 and 3 allowed to confirm that these products, only 1 nt longer in X1702-3 and X1936 than in X2363-2, correspond to 1.3.4f.7 in the first two viruses and to 1.2.4g.7 in the third one.

3 The 367 nt peak seen in X2363-2 may correspond to both 1.2.4c.7 and 1.3.4b.7 *rev* RNAs. Nested PCR using primers recognizing exons 2 and 3 allowed to determine that this peak corresponds to 1.2.4c.7.

**Figure 4:**
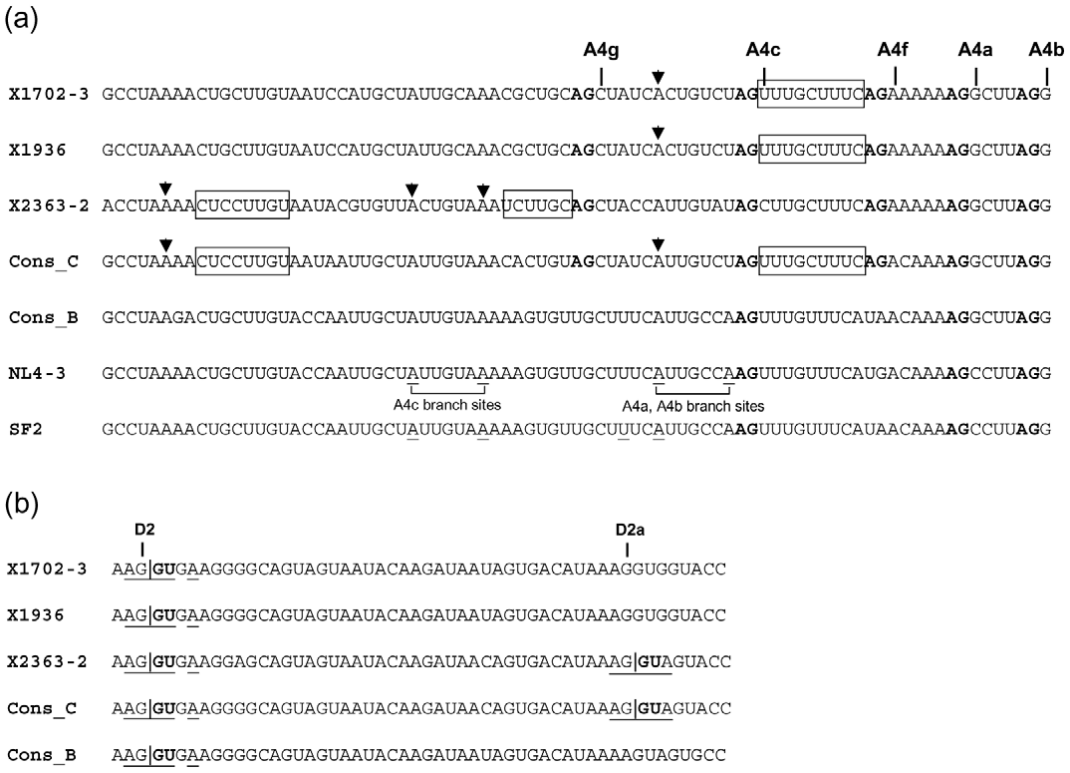
Intronic and exonic sequences surrounding newly identified splice sites in three subtype C isolates. Sequences are aligned with consensuses of subtypes B and C. (a) Sequences surrounding 3'ss A4f and A4g. AG dinucleotides in the intron ends adjacent to splice sites are in bold type. Polypyrimidine tracts potentially used for splicing at A4f and A4g are boxed. The sequences of subtype B NL4-3 and SF2 isolates are on bottom with branch sites previously identified for *rev* RNA splicing [8], [14] underlined. Nucleotides in the subtype C isolates and in the consensus subtype C sequence potentially used as branch points for splicing at A4f and A4g (see main text) are indicated with arrows. (b) Sequences surrounding 5'ss D2 and D2a. Exon-intron borders are signaled with vertical lines. Highly conserved GU dinucleotides at intron ends adjacent to the 5'ss are in bold type. Nucleotides at splice sites potentially pairing with U1 snRNA are underlined.

We examined sequence features surrounding the newly identified splice sites that could explain different splice site usage by the subtype C isolates, compared to subtype B (Fig. 4). The usual elements of the metazoan 3'ss include an AG at the 3′ end of the intron, a branch point site (BPS), usually 18–40 nt upstream of the AG, whose sequence is weakly conserved among mammalians (in humans, the consensus sequence is simply yUnAy, where the underline denotes the branch point, and lowercase pyrimidines are less conserved than the uppercase U and A [21], [22]), and a polypirimidine tract (PPT) downstream of the BPS. All three subtype C isolates have the AG and a PPT with 8 pyrimidines (UUUGUUUUC) (interrupted by a purine, similarly to all HIV-1 3'ss, which are suboptimal due to interspersed purines [11]–[14]) upstream of A4f. All also have an AG 5′-adjacent to A4g, but only X2363-2 has a sequence with 5 pyrimidines (UCUUGC) just upstream of this AG and one with 7 pyrimidines (CUCCUUGU) 34 to 27 nt upstream of A4g, which may contribute to preferential usage of this site in X2363-2 but not in X1702-3 and X1936. Among full-length HIV-1 genomes [23], sequence features consistent with potential usage of A4f and A4g are common in subtype C viruses, but are rare in other subtypes. Thus, among subtype C viruses, the AG adjacent to A4f is found in 86%, and an upstream PPT with 8 pyrimidines in 97% viruses, while the AG adjacent to A4g is found in 87% sequences, with a PPT of 5 pyrimidines just upstream of this AG in 3%, and one of 5 or 6 consecutive pyrimidines within 40 nt upstream of A4g in 60%. In a previous study, four branch points used for generation of *rev* transcripts were identified in the subtype B isolate NL4-3, two for splicing at 3'ss A4a and A4b and two for splicing at 3'ss A4c [14] (Fig. 4a). Three of these branch points were also shown to be used by the subtype B isolate SF2 [8]. One of these BPS, located 20 nt upstream of A4f, could potentially be used for splicing in X1702 and X1936, which have the conserved BPS motif UnA at this site [21], [22]. By contrast, in X2363-2 a C is found at position -2 from the potential branch point which may explain the infrequent use of A4f in this isolate. With regard to A4g, potential BPS are those identified in NL4-3 and SF2 [8], [14], used for splicing at A4c, located 10 and 16 nt, respectively, upstream of A4g. At both sites, the sequence in X2363-2 contains the UnA motif, whereas X1702-3 and X1936 have Cs at position -2 from the branch sites identified in subtype B viruses. If the PPT located 34-27 nt upstream of A4g is the one used for splicing at this site, there is one possible BPS just upstream of this PPT with sequence ACCUAAA, which has 4 consecutive nt complementary to U2 snRNP (underlined) (Fig. 4a), whose base-pairing to the BPS is an important step in mRNA splicing [24], [25]. The sequence analyses therefore may explain differential 3'ss usage for *rev* RNA generation between subtype B and subtype C viruses, and, within subtype C, between different isolates, and suggest the locations of potential BPS used for newly identified 3'ss in subtype C viruses. However BPS locations need to be experimentally determined, as multiple factors in addition to the weakly conserved BPS sequence, including PPT sequence, length, and proximity to the BPS [26]–[29], and the presence of nearby splice enhancer and suppressor elements [16], [30], may influence BPS selection.

With regard to D2a, occasionally used in X2363-2, the sequence is AAG|GUAGUA (the vertical line indicates the exon-intron border), which has 5 potential base-pairings with U1 snRNA (underlined) (Fig. 4b). Previous studies have shown that the strength of a 5'ss correlates with the stability of its interaction with U1 sRNA [31], [32], which for D2a may be similar to D2, which also has 5 potential base-pairings with U1 snRNA. The D2a sequence in X2363-2 coincides with the consensus of most subtypes, except B and H. The subtype B consensus is AAA|GUAGUA, whose predictable weak interaction with U1 snRNA, with only 4 potential discontinuous base-pairings (underlined), may preclude its usage as 5'ss.

## Discussion

This study is the first to analyze splice site usage by viruses of HIV-1 subtype C, which is the most prevalent clade in the HIV-1 pandemic, estimated to represent around 48% global infections [18]. The most notable finding is that subtype C primary isolates, in contrast to subtype B viruses, rarely use 3'ss A4a and A4b for generation of *rev* transcripts, and, instead, they preferentially use two previously unreported 3'ss, designated A4f and A4g, located, respectively, 7 nt upstream of A4a and 14 nt upstream of A4c. Usage of these splice sites is consistent with sequence features commonly found in viruses of subtype C, which frequently contain an AG dinucleotide at the intron's end adjacent to the newly identified splice sites, as well as upstream PPT and sequences with potential to be used as branch points. The infrequent usage of A4a and A4b in subtype C viruses may derive from the linear scanning mechanism for 3'ss recognition [33], whereby the nt after the first AG downstream of the BPS is preferentially selected as splice site. Although the mammalian BPS sequence is highly variable [21], [22], [25], it contains two conserved positions, corresponding to the A at the branch site and the U two nt upstream of it [21], [22], [34]. In two isolates, X1702 and X1936, a potential BPS would be one previously identified in the subtype B isolates NL4-3 and SF2 [8], [14], used for splicing at A4a and A4b, located 20 nt upstream of A4f (Fig. 4a). Although the sequence in X1702 and X1936 at this BPS differs from that of NL4-3 in two nt, the conserved UnA motif is maintained, and at position +1 from the branch site there is one additional potential G-C base-pairing with U2 snRNP, whose complementarity to the BPS has been shown to correlate positively with splicing efficiency [24], [35]. The first AG encountered downstream of this BPS in X1702-3 and X1936 is that immediately upstream of A4f, and this would explain the preferential usage of this splice site over A4a and A4b in these isolates. In the third subtype C isolate, X2363-2, failure to use A4f may derive from sequence changes at the previously mentioned BPS, with C substituting for U at position -2 from the branch site identified in subtype B viruses. The sequence at a second BPS previously identified in NL4-3 for splicing at A4a and A4b, located 6 nt downstream of the previous one, also may fail to function as BPS in X2363-2, because the A used as BPS is substituted for U (Fig. 4a). Although the sequence at a potential branch site may determine its use by the splicing machinery, it is important to note, as stated above, that it is only one factor among others, which also include the PPT sequence, length and proximity to the BPS [26]–[29] and the presence of nearby splice enhancer and suppressor elements [16], [30], contributing to the selection of the BPS, whose actual location needs to be determined experimentally.

The reason A4c is not used more frequently in the analyzed subtype C viruses may derive from weak PPT, which contain 3 or 4 purines interspersed among 8 or 9 pyrimidines. These sequences, in spite of lacking runs of pyrimidines longer than 3 nt, could still act as functional PPT, in accordance with a previous study showing that a stretch of alternating purines and pyrimidines can promote branch point selection [29]. The close proximity of this PPT to the downstream AG [29] and the presence of an exonic splice enhancer (GAR ESE) at exon 5 [36] could also contribute to render this weak PPT functional. In X2363-2, the scanning mechanism selecting A4g as 3'ss would also explain the infrequent usage of A4c and other downstream 3'ss.

Occasional use of a new 5'ss, designated D2a, located 41 nt downstream of D2, was also observed in one subtype C isolate, X2363-2. Usage of D2a is also consistent with sequences present in this isolate and in most subtype C viruses, which have greater complementarity with U1 snRNA at this site relative to subtype B viruses. In addition, the usage of D2a as an alternative to D2 in subtype C may be favored by the fact that D2 is a suboptimal 5'ss [15]. Its less frequent usage relative to D2 may derive from the scanning mechanism proposed for recognition of the 5'ss, whereby among several consecutive potential sites, the 5′-most site is usually selected [37].

With the newly identified sites, seven 3'ss have been reported to be used in HIV-1 for *rev* RNA generation, which, in addition to the commonly used A4a, A4b, and A4c, also include A4d, located 5 nt upstream of A4a, reported in the subtype B isolate SF2 and the group O virus ANT70C [8] [and also preferentially used by one additional subtype B primary isolate studied by us (unpublished data)], and A4e, located 1 nt upstream of A4a, reported in ANT70C [8] (and, according to the presence of an intronic AG dinucleotide adjacent to the A4e site, also predicted to be used by most subtype F and CRF02_AG viruses). Such multiplicity of 3'ss used for *rev* RNA generation may derive from the facts that *rev* 3′ splice sites are located in the first coding exon of Tat, which is one of the most variable HIV-1 proteins [38], and that HIV-1 replication is absolutely dependent on Rev, whose absence cannot be compensated by viruses from other infected cells, as occurs with Tat, which can be secreted extracellularly and activate HIV-1 transcription in neighboring cells [39].

Previously reported *in vitro* biological features which may differ between HIV-1 subtypes include the response of the transcriptional promoter to tumor necrosis factor-alpha [40]–[44], replicative capacity [45], [46], use of coreceptors [47]–[51], and activity of reverse transcriptase [52]. The results here reported add one more biological feature in which HIV-1 subtypes may differ, which is the usage of RNA splice sites.
